# Impact of Vapor Barrier on Moisture Content of Fiberboard Insulation in Log Structure

**DOI:** 10.3390/polym13244282

**Published:** 2021-12-07

**Authors:** Stanislav Jochim, Róbert Uhrín, Jarmila Schmidtová, Pavol Sedlák, Dominika Búryová, Katarína Střelcová

**Affiliations:** 1 Department of Wood Structures, Technical University in Zvolen, 96001 Zvolen, Slovakia; robert.uhrin@tuzvo.sk (R.U.); sedlak_pali@yahoo.com (P.S.); buryova@tuzvo.sk (D.B.); 2 Department of Mathematics and Descriptive Geometry, Technical University in Zvolen, 96001 Zvolen, Slovakia; jarmila.schmidtova@tuzvo.sk; 3 Department of Natural Environment, Technical University in Zvolen, 96001 Zvolen, Slovakia; strelcova@tuzvo.sk

**Keywords:** timber structures, loadbearing log wall, moisture content of insulation, fiberboard insulation, vapor barrier

## Abstract

The paper is focused on a verification of the moisture content of fiberboard insulations in the multilayer loadbearing log wall designed with and without the vapor barrier. Experimental verification was done using a sample of the multilayer loadbearing log wall built in a research timber structure building under in-situ conditions. Indoor properties of the building met conditions for human occupancy. The experiment was performed for 2 years and 3 months. Aims of the fiberboard insulations moisture content verification in the walls were to verify the effect of vapor barrier in various periods of the year and verify excessive moisture in the fiberboard insulations, which is undesirable in terms of biodegradation. The results of measuring the moisture content showed that after a certain period, the difference of insulation moisture content in the wall including and excluding vapor barrier is negligible, as well as other results and conclusions for designing the composition of multilayer loadbearing log walls.

## 1. Introduction

Log structures are unquestionably attractive and architecturally interesting structures for human occupancy, year-round lodging and other purposes. Historically, log walls have been designed as a single layer made of lumber, with a width of 200 to 400 mm. Joints of log walls and joinery details were sealed to prevent thermal loss of the building due to air leakage. They were erected in various ways with various effects on air tightness. Significant thermal loss results from a heat transfer through the log wall. Design of these structures became insufficient in terms of increasing thermal performance demands due to energy saving and environment impact. 

In order to decrease the heat transfer, a multilayer log wall is designed using various insulations on the interior wall side, leading to improvement of the thermal performance of the log wall. Application of the vapor barrier layers of various effectivity can prevent interior air humidity transfer into the structure and water vapor condensation inside the structure, thus eliminating conditions favorable for the lumber biodegradation. Thermal insulation applied between the studs and in the service cavity can be organic or inorganic based, with various heat transfer, diffusion and other properties. The interior is finished with a timber cladding or mineral board and drywall. Design aim of multilayer log wall is to achieve required thermal performance properties such as heat transfer coefficient, superficial temperature excluding the risk of mold growth and superficial temperature in the wall excluding or limiting water vapor condensation and others.

Thermal performance properties are determined by calculations of various methods based on a fundamental material physical property. However, methods of calculation cannot reliably cover other essential complex material properties, e.g., processes of moisture transfer, material moisture content in the structure, critical dew points in loadbearing structures. Moreover, these methods do not describe complex influence of climate-related nonstationary conditions affecting a structure. Therefore, experimental verifying under in-situ conditions using a measuring tool to record the climate conditions and an oven-dry method to verify the moisture content of the materials must be performed for measurement accuracy verification of material moisture content and critical dew points in multilayer loadbearing walls. 

Research on the thermal performance properties is conducted simultaneously in specific software and experimentally in-situ. The influence of vapor barrier equivalent diffuse thickness and the width of an insulation on mold growth risk in log structures was investigated in Alev et al. [1]. The paper published by Conroy et al. [2] verified the hygrothermal performance of a post and beam building designed to meet the International Passive House Standard both in-situ and via software WUFI^®^. Geving et al. [3] investigated the moisture conditions in several samples of the wood frame wall insulated with loose-fill and batt wood fiber as well as batt glass wool in variations with wind barrier, vapor barrier, vapor retarder or no barrier. Moreover, Glass et al. [4] verified the interior vapor retarder on the performance of six configurations of energy efficient walls at elevated levels of interior relative humidity, Goodhew et al. [5] verified the moisture content of the wood-discs placed in external straw-bale walls of a case study building. Volf et al. [6] investigated physical properties of the selected natural insulating materials and tested possible mold growth risk in laboratory conditions. The vapor barrier influence on the materials moisture content and water vapor condensation in timber structures is also verified in [7,8,9,10,11,12,13]. Materials biodegradation in timber structures is verified in [14,15,16].

The aim of this paper is medium-term experimental moisture content verification of the fiberboard insulations in the two samples of multilayer loadbearing log walls, including and excluding vapor barrier. 

## 2. Materials and Methods

### 2.1. Materials—Loadbearing Log Structures

**Figure 1:**
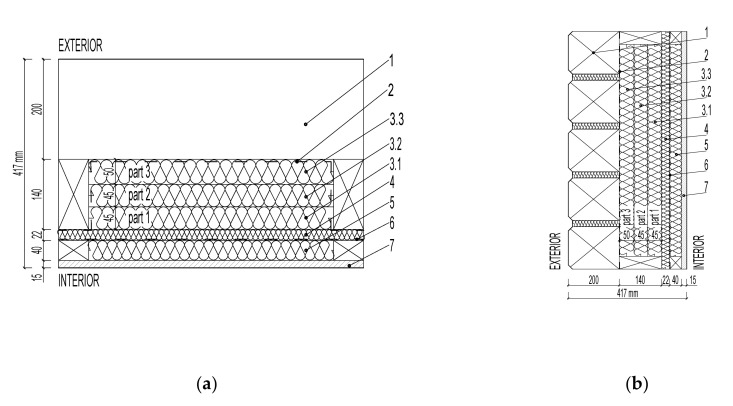
Log Wall LW1 with Vapor Barrier: (**a**) floor plan; (**b**) section. (1) Log Lumber; (2) House wrap ISOCELL OMEGA 180; (3.1) Fiberboard Insulation STEICO Flex, part 1; (3.2) Fiberboard Insulation STEICO Flex, part 2; (3.3) Fiberboard Insulation STEICO Flex, part 3; (4) Fiberboard Insulation STEICO Universal; (5) Fiberboard Insulation STEICO Flex; (6) Vapor Barrier ISOCELL.

**Figure 2:**
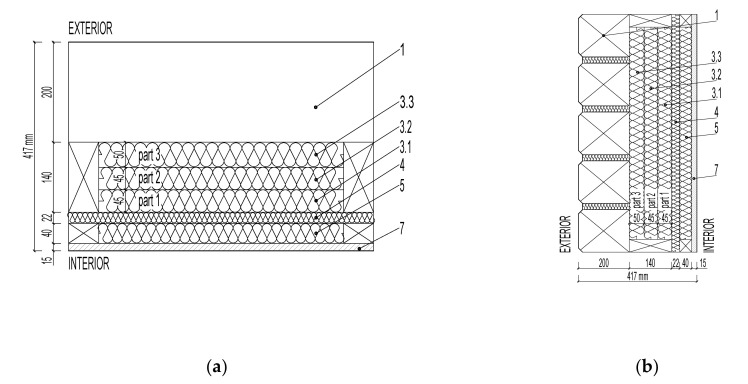
Log Wall LW2 without Vapor Barrier: (**a**) floor plan; (**b**) section. (1) Log Lumber; (3.1) Fiberboard Insulation STEICO Flex, part 1; (3.2) Fiberboard Insulation STEICO Flex, part 2; (3.3) Fiberboard Insulation STEICO Flex, part 3; (4) Fiberboard Insulation STEICO Universal; (5) Fiberboard Insulation STEICO Flex; (7) Interior Cladding.

Two samples of multilayer loadbearing log walls were designed for the experimental moisture content verification of the fiberboard insulation:

- Wall LW1 with paper vapor barrier—Figure 1.
- Wall LW2 without paper vapor barrier—Figure 2.

Thermal performance properties of the walls are shown in Table 1.

### 2.2. Fiberboard Insulation Used in Verification

The moisture content of the fiberboard insulations was verified in the walls LW1 and LW2 in Figure 1 and Figure 2:

1. STEICO Flex with a width of 40 mm, thermal conductivity coefficient λ = 0.038 W·m^−1^·K^−1^
2. STEICO Universal with a width of 22 mm, λ = 0.05 W·m^−1^·K^−1^·
3. STEICO Flex with a width of 140 mm, λ = 0.038 W·m^−1^·K^−1^, divided into 3 parts, part 1 width 45 mm, part 2 width 45 mm, part 3 width 50 mm, for more accurate measurement of the moisture distribution.

### 2.3. Water Vapor Barrier and House Wrap Used for the Verification

Paper water vapor barrier ISOCELL Oko Natur was used, the value of equivalent diffuse thickness S_d_ = 6.45 m, diffusion resistance factor µ = 32,250.

House wrap OMEGA 180 was used with the value of equivalent diffuse thickness S_d_ = 0.025 m, diffusion resistance factor µ = 28.

### 2.4. In-Situ Conditions and Period of Verification

**Figure 3:**
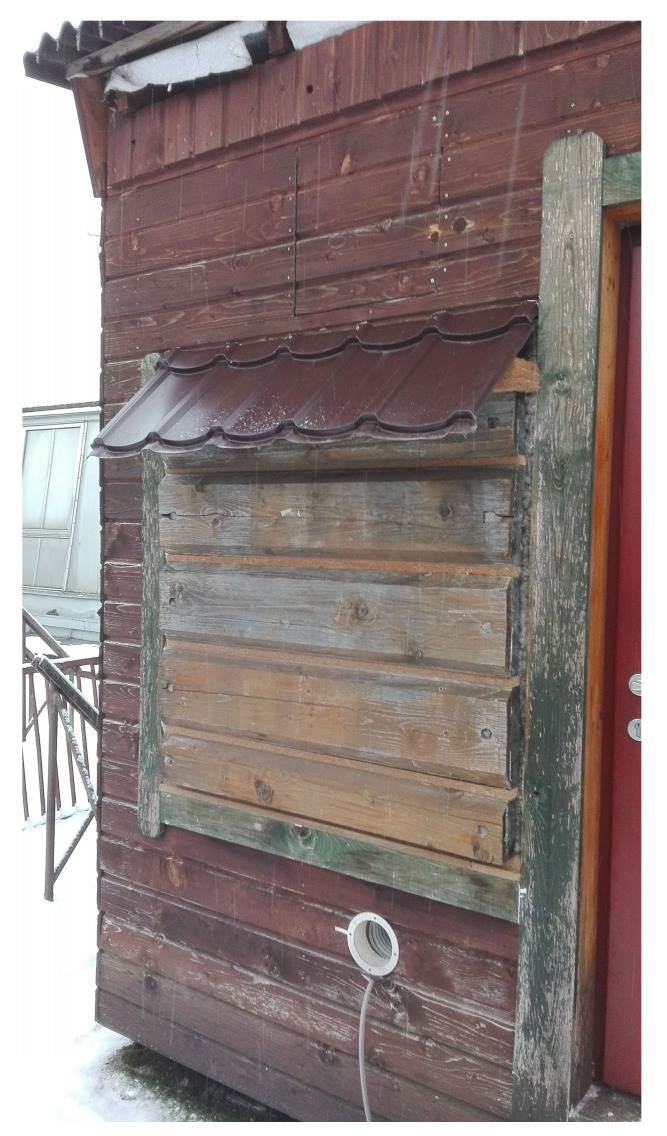
Log Wall LW1 and LW2 Installed in the Research Timber Structure, Exterior.

**Figure 4:**
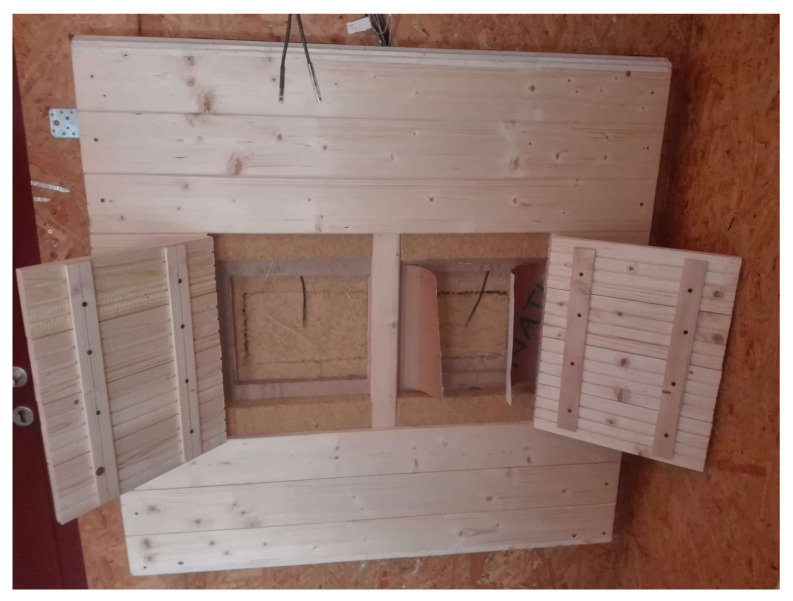
Log Wall LW1 and LW2 Installed in the Research Timber Structure, Interior.

**Figure 5:**
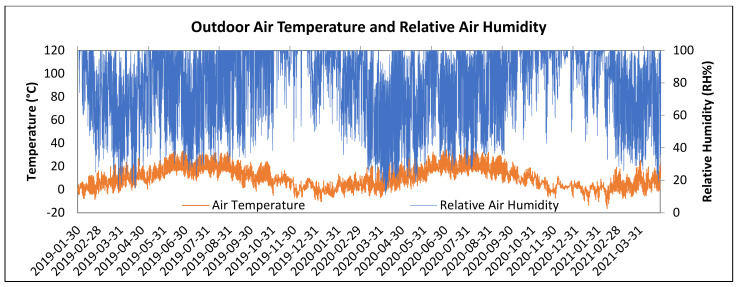
Outdoor Air Temperature and Relative Air Humidity during the Recorded Period.

**Figure 6:**
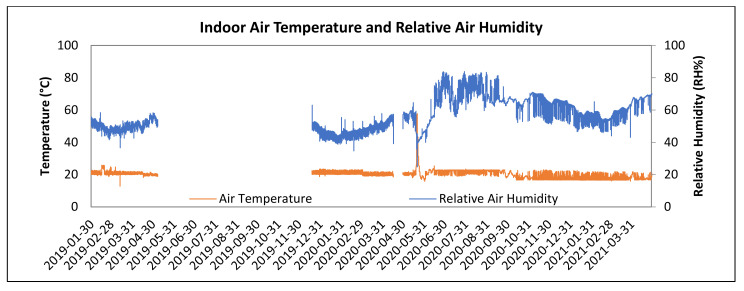
Indoor Air Temperature and Relative Air Humidity during the recorded period.

LW1 and LW2 walls were installed into the north-facing wall of the research timber structure as shown in Figure 3 and exposed to an exterior weather. Interior conditions simulated human occupancy. Interior side of the walls is shown in Figure 4. In-situ conditions were recorded by measuring tools: air temperature and relative air humidity at 1-h intervals. The recorded conditions are shown in Figure 5 and Figure 6.

The experiment is in Zvolen, Slovak Republic, altitude 300 MSL, with a temperature inversion frequently occurring in spring and autumn. The period of verification was 2 years and 3 months, from 30 January 2019, to 3 May 2021.

Interior conditions for human occupancy were set at the air temperature of 20 ± 3 °C and relative air humidity 50 ± 10%. Higher relative air humidity, 60 to 80% was reached in humid rooms such as bathrooms, laundries, kitchen, etc. During the period from May 7 to December 12, 2019, an outage occurred causing data to be rewritten and deleted. Similar outages happened during the year 2020 as well.

### 2.5. Climatic Conditions in the Multilayer Loadbearing Log Structures

**Figure 7:**
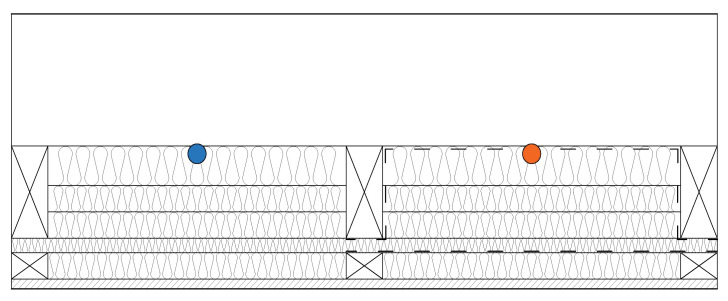
Sensors Layout in the Walls LW1 (orange, right) and LW2 (blue, left).

Climatic conditions in the walls were recorded by the measuring tool AHLBORN ALMEMO 5690 and sensors AHLBORN FHAD46C2 recording air temperature, relative air humidity and air pressure at 1-h intervals. The layout of the sensors is shown in Figure 7. 

### 2.6. Method of the Moisture Content Verification

The oven-dry method was used to verify moisture content of the insulations in the walls in accordance with the standard EN 322:1995[18]. Weighing intervals were 2 to 4 weeks. Scale used for weighing method was RADWAG WLC 2/A2.

### 2.7. Evaluation Method of the Aims of the Paper

Firstly, verification of the vapor barrier effectivity on the moisture content of fiber-board insulation based on the results comparison of the oven-dry method during the heating season and rest of the year. The first hypothesis: it is assumed that the average fiberboard insulation moisture content in the wall with the vapor barrier will be different (percentual change by 5% or higher) during the heating season and the rest of the year compared to fiber-board insulation without the vapor barrier. Thus, the effect of the vapor barrier will be significant. 

Verification of the vapor barrier effectivity on the insulation moisture content was evaluated by specifying the heating season and the result comparison of percentual change of the oven-dry method.

The heating season is a period of the year during which it is necessary to provide heating in buildings due to colder weather. The heating season was set:

- The heating season 2019 (HS19): from 30 January 2019 to 30 April 2019
- The heating season 2020 (HS20): from 1 November 2019 to 30 April 2020
- The heating season 2021 (HS21): from 1 November 2020 to 30 April 2021

The non-heating season is a period of the year during which it is not necessary to provide heating in building.

- The non-heating season 2019 (nHS19): from 1 May 2019 to 30 October 2019
- The non-heating season 2020 (nHS20): from 1 May 2020 to 30 October 2020

Secondly, verification of the undesirable excessive moisture due to the risk of biodegradation in fiberboard insulations in both fragments of the log walls. The second hypothesis: it is assumed that the critical average moisture content exceeding 20% causing the risk of biodegradation with mold and wood decaying fungi will not be reached in the fiberboard insulations of the log wall with and the wall without vapor barrier.

Verification of the undesirable excessive moisture increasing the risk of insulation biodegradation was evaluated based on the average moisture content of the fiberboard insulation STECO Flex part 3 as shown in Figure 1 (3.3) and Figure 2 (3.3), near the loadbearing log. The average moisture content was compared in the periods when relative air humidity in the walls reached 70% or more. 

### 2.8. Statistical Method for Evaluation of the Aims of the Paper

The analyses were carried out using statistical software STATISTICA 12. The fact whether the variability of the outcomes is due to chance or to effect of factors (or their interaction) was tested applying a two-way ANOVA. This technique enables researchers to estimate how the mean of a quantitative (dependent) variable is changed according to the levels of two categorical (independent) variables called factors. One sample T-test was used to test population mean against a “limit value”, which is a known value from industry standards. In all tests, a 5% level of significance was used.

## 3. Results

Outdoor air temperature and the relative air humidity values were recorded during the monitored period as shown in Figure 5.

Interior air temperature was maintained at 20 ± 3 °C, and the relative air humidity at 50 ± 10%, conditions matching human occupancy in accordance with the national standard as shown in Figure 6.

During the period from May 7 to December 12, a power cut caused reset of the settings in measuring instrument without indication of error, which caused data to be rewritten and ultimately deleted. A similar short-term outage happened during the year 2020.

**Figure 8:**
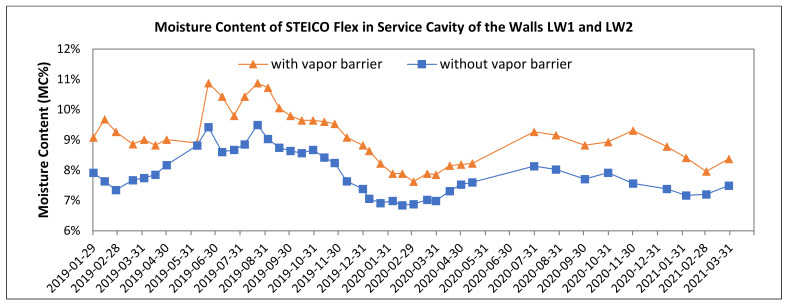
The Moisture Content of the Fiberboard Insulation in Service Cavity.

**Figure 9:**
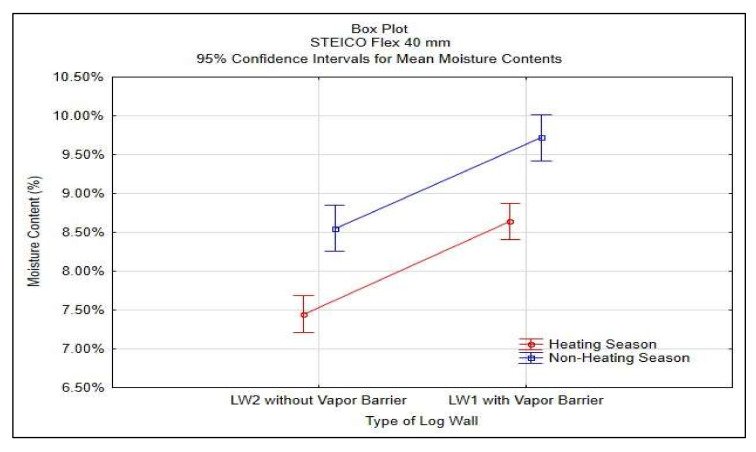
95% Confidence Intervals for Mean Moisture Contests of STEICO Flex width 40 mm.

**Figure 10:**
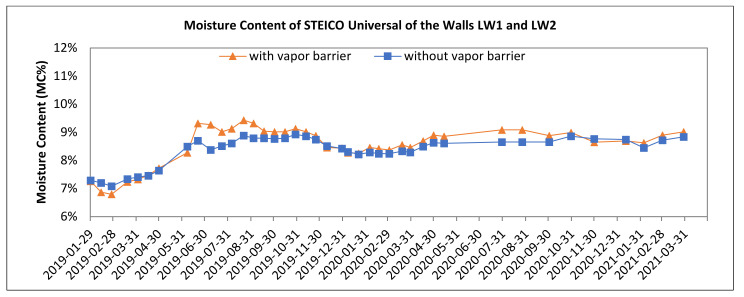
The Moisture Content of Fiberboard Insulation following Vapor Barrier.In the wall LW1 with the vapor barrier the insulation STEICO Universal reached slightly higher moisture content during the spring and the summer, it was especially due to reversed vapor transfer, i.e., outdoor humidity moves indoors entering the wall. The vapor barrier prevents the vapor transfer, and it was accumulated in the insulation situated in front of the barrier.

**Figure 11:**
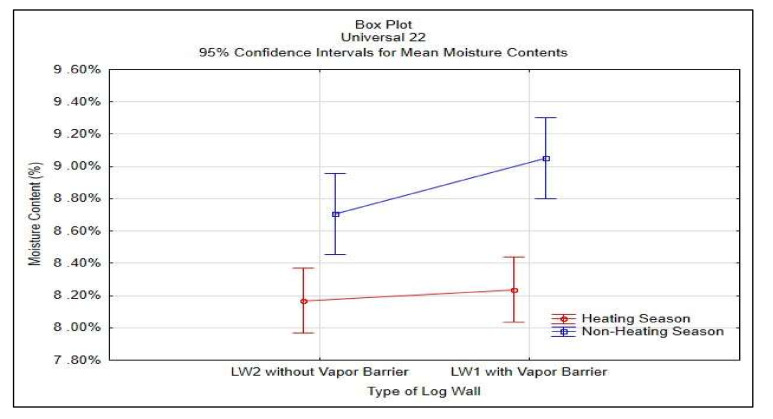
95% Confidence Intervals for Mean Moisture Contests of STEICO Universal 22 mm.

**Figure 12:**
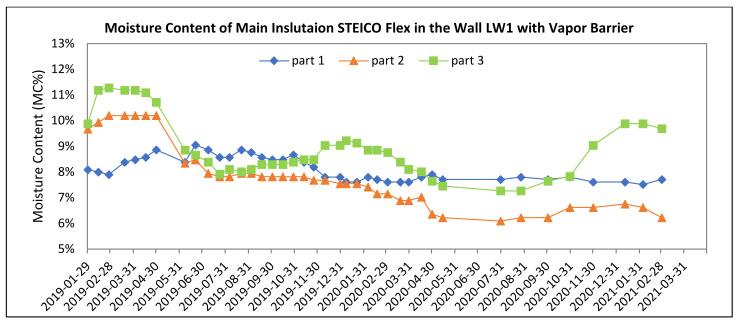
The Moisture Content of the Fiberboard Insulation between Studs in the Wall with Vapor Barrier.

**Figure 13:**
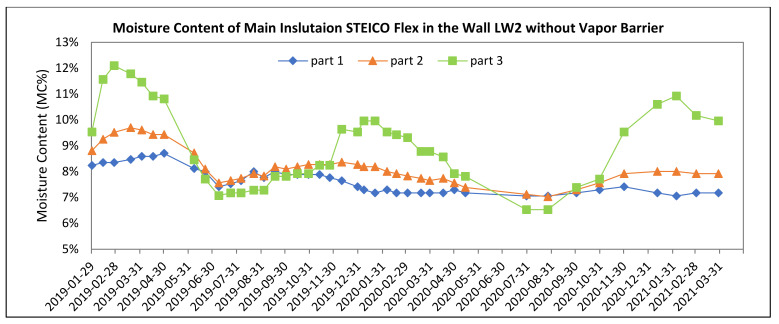
The Moisture Content of the Fiberboard Insulation between Studs in the Wall without Vapor Barrier.

Figure 8, Figure 9, Figure 10, Figure 11, Figure 12 and Figure 13 present the moisture content in the fiberboard insulations measured by the oven-dry method during the period of verification.

The insulation STEICO Flex situated in a service cavity in the wall LW1 with the vapor barrier reached higher moisture content than the same insulation situated in the wall without vapor barrier. The vapor barrier preventing the vapor transfer from the interior to the wall can be considered the reason. This humidity was accumulated in the service cavity in front of the barrier. 

Statistics analysis was done in STATISTICA^®^ software, results shown in Table 2 and Figure 9.

Statistical analysis was done in STATISTICA^®^ software, results shown in Table 3 and Figure 11.

**Figure 14:**
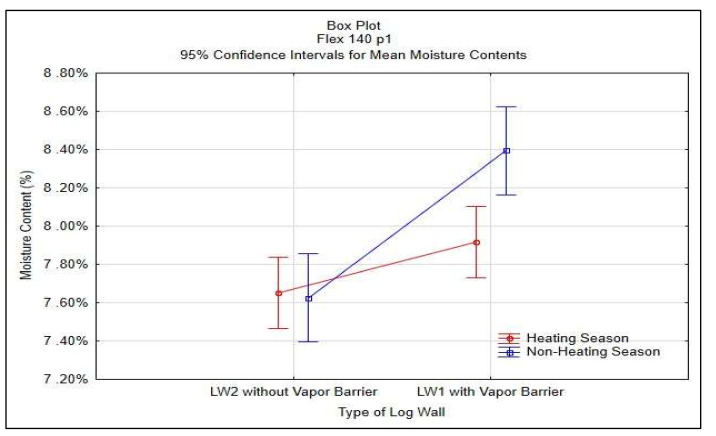
95% Confidence Intervals for Mean Moisture Contests of STEICO Flex part 1.

Statistical analysis of STEICO Flex part 1 was done in STATISTICA^®^ software, results shown in Table 4 and Figure 14.

**Figure 15:**
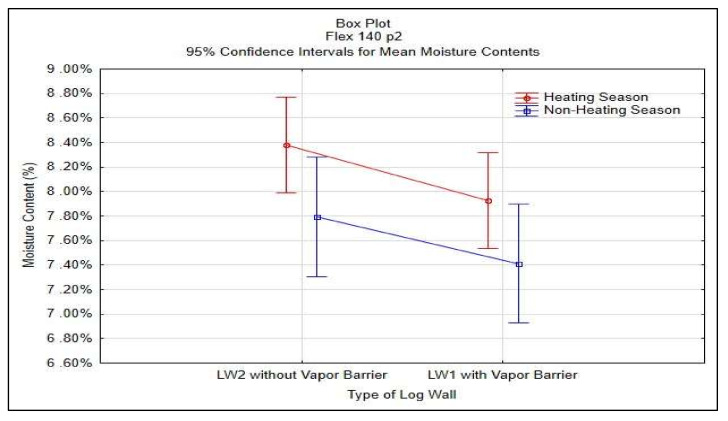
95% Confidence Intervals for Mean Moisture Contests of STEICO Flex part 2.

Statistical analysis of STEICO Flex part 2 was done in STATISTICA^®^ software, results shown in Table 5 and Figure 15.

**Figure 16:**
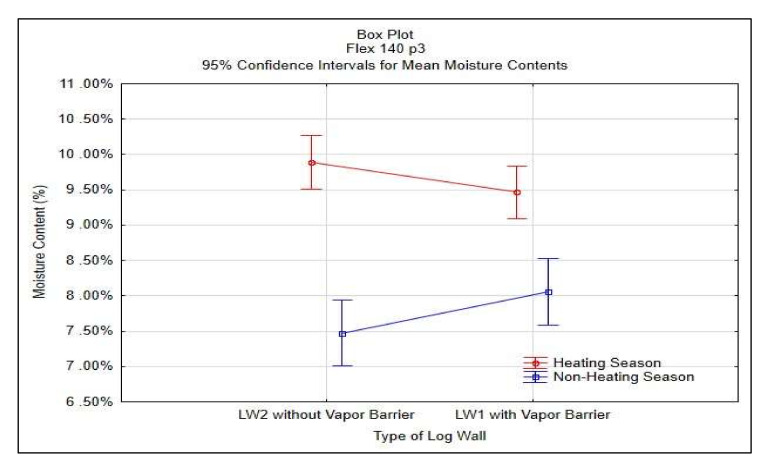
95% Confidence Intervals for Mean Moisture Contests of STEICO Flex part 3.

Statistical analysis of STEICO Flex part 3 was done in STATISTICA^®^ software, results shown in Table 6 and Figure 16.

Statistical analysis of factor analyzed influence on insulation moisture content was done in STATISTICA^®^ software, results shown in Table 7.

**Figure 17:**
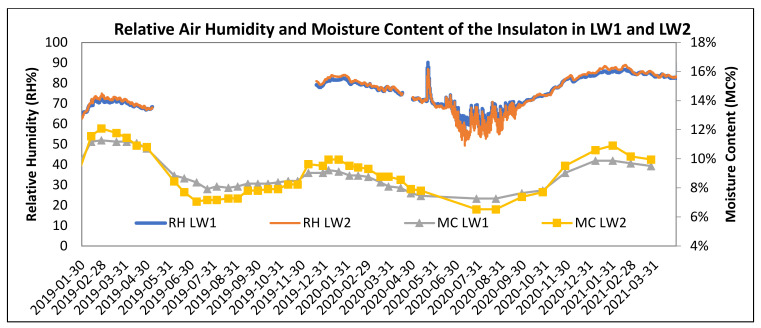
The Moisture Content of the Fiberboard Insulation STEICO Flex part 3 between Studs and Recorded Relative Air Humidity in the Walls.

Figure 17 shows moisture content of the STEICO Flex part 3 and relative air humidity in the walls.

**Figure 18:**
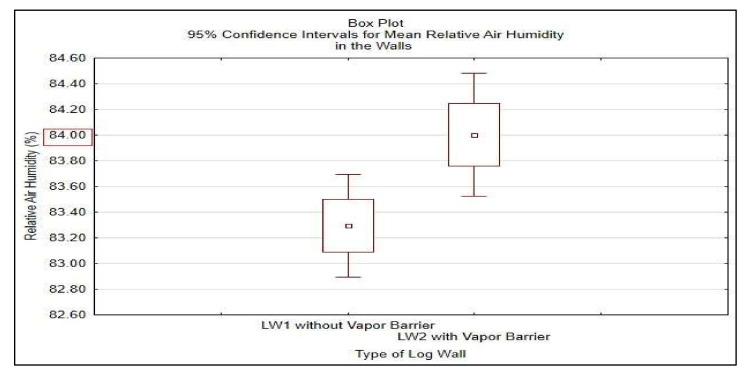
95% Confidence Intervals for Mean Relative Air Humidity in the Walls in the HS21.

**Figure 19:**
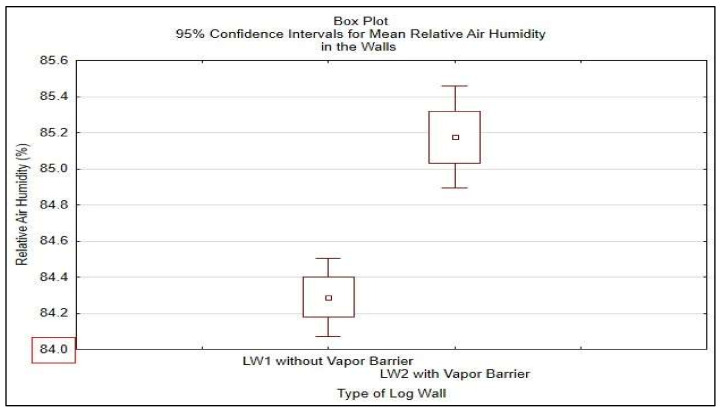
95% Confidence Intervals for Mean Relative Air Humidity in the Walls from 1 December 2020 to 30 April 2021.

Statistical analysis was done in STATISTICA^®^ software, results shown in Table 8 and Figure 18 and in Table 9 and Figure 19.

## 4. Discussion

The significance of the vapor barrier effectivity on the moisture content of fiberboard insulations in the walls LW1 and LW2 during the season of the year is proved if the percentual change in the average moisture content is 5% or higher. The significant differences were tested on the alpha level 0.05. Subsequently, 95% confidence intervals for means of moisture content were calculated. The results of statistics analysis are shown in Table 7. The moisture content of the insulations in the walls LW1 and LW2 are shown in Figure 8, Figure 10, Figure 12, Figure 13. The results of comparison are:

- The average moisture content of the STEICO Flex insulation in the service cavity in the wall LW1 compared to the wall LW2 has:
  ◦ Higher average moisture content, percentual change in HS19 of 14.75%,
  ◦ Higher average moisture content, percentual change in nHS19 of 12.72%,
  ◦ Higher average moisture content, percentual change in HS20 of 12.53%,
  ◦ Higher average moisture content, percentual change in nHS20 of 8.11%,
  ◦ Higher average moisture content, percentual change in HS21 of 13.47%.

The results indicate that the vapor barrier effectivity on the insulation moisture content was significant, and the insulation moisture content in the service cavity of the wall LW1 is higher than that of the wall LW2. 

The results in Table 2 and Figure 9 confirm the significance of differences of the vapor barrier effectivity on the insulation moisture content and at the same time confirm the significance of the effect of heating and non-heating season on the insulation moisture content.

- The average moisture content of the insulation following the vapor barrier STEICO Universal in the wall LW1 compared to the wall LW2 has:
  ◦ Lower average moisture content, percentual change in HS19 of −1.45%,
  ◦ Higher average moisture content, percentual change in nHS19 of 5.00%,
  ◦ Higher average moisture content, percentual change in HS20 of 1.65%,
  ◦ Higher average moisture content, percentual change in nHS20 of 3.82%,
  ◦ Higher average moisture content, percentual change in HS21 of 1.16%.

The results indicate that the vapor barrier effectivity on the insulation moisture content was significant in HS19; otherwise, the effectivity proved not significant.

The results in Table 3 and Figure 11 do not confirm the significance of differences of the vapor barrier effectivity on the insulation moisture content but confirm the significance of the effect of heating and non-heating season on the insulation moisture content.

- The average moisture content of the insulation between studs STEICO Flex, part 1 in the wall LW1 compared to the wall LW2 has:
  ◦ Lower average moisture content, percentual change in HS19 of −1.73%,
  ◦ Higher average moisture content, percentual change in nHS19 of 10.31%,
  ◦ Higher average moisture content, percentual change in HS20 of 5.3%,
  ◦ Higher average moisture content, percentual change in nHS20 of 7.9%,
  ◦ Higher average moisture content, percentual change in HS21 of 6.5%.

The results indicate that the vapor barrier effectivity on the insulation moisture content was not significant in HS19; otherwise, the effectivity proved significant.

The results in Table 4 and Figure 14 confirm the significance of differences of the vapor barrier effectivity on the insulation moisture content and at the same time confirm the significance of the effect of heating and non-heating season on the insulation moisture content.

- The average moisture content of the insulation between studs STEICO Flex, part 2 in the wall LW1 compared to the wall LW2 has:
  ◦ Higher average moisture content, percentual change in HS19 of 6.87%,
  ◦ Higher average moisture content, percentual change in nHS19 of 0.63%,
  ◦ Lower average moisture content, percentual change in HS20 of −8.81%,
  ◦ Lower average moisture content, percentual change in nHS20 of −17.10%,
  ◦ Lower average moisture content, percentual change in HS21 of −19.54%.

The results indicate that the vapor barrier effectivity on the insulation moisture content was not significant in HS19; otherwise, the effectivity proved significant.

The results in Table 5 and Figure 15 do not confirm the significance of differences of the vapor barrier effectivity on the insulation moisture content but confirm the significance of the effect of heating and non-heating season on the insulation moisture content.

- The average moisture content of the insulation between studs STEICO Flex, part 3 in the wall LW1 compared to the wall LW2 has:
  ◦ Lower average moisture content, percentual change in HS19 of −2.15%,
  ◦ Higher average moisture content, percentual change in nHS19 of 9.64%,
  ◦ Lower average moisture content, percentual change in HS20 of −3.32%,
  ◦ Higher average moisture content, percentual change in nHS20 of 5.75%,
  ◦ Lower average moisture content, percentual change in HS21 of −4.42%.

The results indicate that the vapor barrier effectivity on the insulation moisture content is undetermined. 

The results in Table 6 and Figure 16 do not confirm the significance of differences of the vapor barrier effectivity on the insulation moisture content and at the same time confirm the significance of the effect of heating and non-heating season on the insulation moisture content.

The vapor barrier effectivity on the fiberboard insulations moisture content in the walls LW1 and LW2:

- Is significant in the insulation STECO Flex, shown in Figure 1 (5) and Figure 2 (5), the vapor barrier prevents and slows down the moisture transfer from the interior into the wall
- In general, is not significant in the insulation STEICO Universal, shown in Figure 1 (4) and Figure 2 (4),
- In general, is significant in the insulation STEICO Flex part 1, shown in Figure 1 (3.1) and Figure 2 (3.1); the moisture content of the insulation in the wall LW1 is generally higher than the wall LW2,
- In general, is significant in the insulation STEICO Flex part 2, shown in Figure 1 (3.2) and Figure 2 (3.2); the moisture content of the insulation in the wall LW1 decreases in time compared to the insulation in the wall LW2,
- In general, the significance in the insulation STEICO Flex part 3, shown in Figure 1 (3.3) and Figure 2 (3.3), cannot be determined; the moisture content of the insulation in the wall LW1 and LW2 oscillates depending on the weather conditions, probably caused by imperfect airtightness of the gaps in the loadbearing log wall.

Undesirable excessive moisture is considered for the average moisture content of 20% lasting more than 2 weeks continuously. The verification of the undesirable excessive moisture increasing the risk of biodegradation is done at the border of the insulations STEICO Flex part 3 and the loadbearing log wall. The recorded values of the relative air humidity, shown in Figure 17, presuppose conditions favorable for the mold growth and wood decaying fungi at this border, particularly when relative air humidity reaches 70% or more. The statistics analysis results are shown in Table 8 and Figure 18. Average moisture content of the insulation STEICO Flex part 3 in the wall LW1 and LW2 reached during this period are compared below.

The relative air humidity in the walls reached 70% or more:

- from 29 January to 16 April 2019, the insulation in LW1 reached the average moisture content of 10.98% and the insulation in LW2 reached the average moisture content of 11.23%;
- from 10 December 2019 to 15 June 2020, the insulation in LW1 reached the average moisture content of 8.51% and the insulation in LW2 reached the average moisture content of 8.90%;
- from 1 October 2020 to 29 March 2021, the insulation in LW1 reached the average moisture content of 9.05%, and the insulation in LW2 reached the average moisture content of 10.38%.

The results indicate that average moisture content of the insulations STEICO Flex part 3 in both walls LW1 and LW2, under the recorded weather and indoor conditions did not exceed MC = 20%, and thus, no risk of the biodegradation appeared in the walls. The insulation in the wall LW2 had in general insignificantly higher average moisture content compared to the wall LW1.

To confirm these results, relative air humidity in the walls was further investigated from 1 December 2020 to 30 April 2021. Borderline relative air humidity favorable for biodegradation was set to RH = 84% in accordance with the national standard in this period, and Student’s T-test was done. The results shown in Table 9 and Figure 19 confirm mean relative air humidity in the LW1 of 84.29% and in the LW2 of 85.18%. The insulation STEICO Flex part 3 in LW1 reached the average moisture content of 9.6% and the insulation in LW2 reached the average moisture content of 10.2%. The average moisture content of the insulation STEICO Flex part 3 in both walls LW1 and LW2 did not exceed 20%, and no risk of biodegradation appeared in the walls. 

Moisture contents of the materials in timber structures under in-situ conditions are described in the paper of Conroy [2]. In the paper, the authors verified hygrothermal performance of a post and beam building designed to meet the International Passive House Standard both in-situ and via WUFI^®^ software. Thicker walls dry out slower, and humidity could accumulate according to the paper conclusions. However, the in-situ results show no accumulation of the humidity in the wall and no risk of biodegradation. Similar conditions of slow drying out of the walls could appear in the walls LW1 due to the combined effect of the house wrap and the thickness of the log and in LW2 due to the thickness of the log. However, gaps between logs are filled with the insulation STEICO Flex with a width of 30 mm, which is vapor permeable, thus allowing the walls to dry out more intensively. It supports the results of the moisture content of STEICO Flex part 3 in the non-heating season when the moisture content is lower than in the heating season and the humidity does not accumulate in the insulation. Results of Geving et al. [3] indicate slightly better performance of the loose fill fiber insulation compared to batt fiber insulation and positive influence on the moisture conditions when 50 mm wood fiber board was used as a wind barrier. Positive influence was proven using so-called smart vapor barrier. The results are comparable to this work, specifically in measured values of relative air humidity in the samples. In the paper of Glass [4], interior vapor retarder effect on the performance of six configurations of energy efficient walls at elevated levels of interior relative humidity is verified. The results prove that oriented strand board used in configuration of the wall without vapor retarder and interior drywall with latex paint had higher moisture content compared to the configuration with vapor retarder. The consequence of the higher moisture content resulted in mold growth on the OSB, which is undesirable. Volf et al. [6] investigated physical properties of the selected natural insulating materials and tested possible mold growth risk in laboratory conditions and determined that natural insulation has comparable thermal properties to common building insulation materials. Although there was no mold growth observed in samples, fungi spores could be present in natural insulation; therefore, proper and safe constructing design is extremely important. Volf et al. [6] used fiberboard with a density of 51.5 kg·m^−3^ and measured relative air humidity of 85%, and the moisture content was approximately 8.5%. Fiberboard used in this work had a density of 60 kg·m^−3^, and the average moisture content of the insulation in LW2 was 10.2%. It is assumed that higher moisture content in the insulation was caused by higher density and higher relative air humidity in the wall of 89% in HS21. In comparison with this work, there is no mold growth in the wall LW2 because of redistribution of the moisture in the insulation. Alev [1] simulated the risk of mold growth in log structure using software WUFI. The results are principles for designing log structures, such as appropriate choice of vapor barrier and width of the insulation. Due to different timber structures, verified materials, weather and geographical conditions and the period of verification in the papers of Conroy [2] and Glass [4], these results cannot be compared. Moisture content verifying methods and climate conditions both outdoors and indoors are identical.

The research of the fiberboard insulation moisture content in log walls LW1 and LW2 brought interesting new knowledge, for example the initial assumption that the vapor barrier effect would be significant for every insulation in the wall proved wrong, the vapor barrier effect proved significant in STEICO Flex in the service cavity and STEICO Flex part 1 and part 2, while the effect proved not significant in STEICO Universal and STEICO Flex part 3. Research into vapor barrier effect on the insulation based on the type and physical properties of insulation and different position of the vapor barrier in the wall is suggested. 

## 5. Conclusions

Based on the aim of the paper to verify the vapor barrier effectivity on the moisture content of the wall insulations and to verify undesirable excessive moisture due to the risk of biodegradation in the fiberboard insulations following results can be presented:

- Vapor barrier effect is proved to be significant on the insulation STEICO Flex width in the service cavity, STEICO Flex part 1 and 2 between the studs; percentual change of the average moisture content in the insulations in the wall LW1 is 5% or higher.
- Vapor barrier effect is not significant on the insulation STEICO Universal; percentual change of the average moisture content in the insulation in the wall LW1 is in general 5% or lower than in the wall LW2 during seasons of the year.
- The significance of the vapor barrier effect on the insulation STEICO Flex part 3 between studs cannot be determined due to the oscillating moisture content of the insulation depending on the weather conditions, probably caused by imperfect airtightness of the gaps in the loadbearing log wall.
- The average moisture content of the insulations STEICO Flex part 3 in both walls LW1 and LW2, under the recorded weather and indoor conditions did not exceed 20%; therefore, the risk of the mold growth and biodegradation by wood decaying fungi is excluded.

Conclusions for the practice are:

- Applying vapor barrier in peripheral log walls of a log structure is favorable for the positive effect on the moisture content of the insulations, which proved significant and for the positive effect on airtightness, which prevents humidity transfer from the interior into the wall structure causing lower moisture content and lower risk of biodegradation.
- Spaces between horizontal logs should be properly filled to prevent undesired effects of the wind and keep the spaces open for diffusion of the water vapor.
- To design a log structure without any vapor barrier requires a professional approach and safe moisture content verification of the design.

## Figures and Tables

**Table 1:** Thermal performance properties of the sample walls LW1 and LW2.

Wall	Heat Transfer Coefficient	Thermal Resistance	Superficial Temperature	The All-Year Amount of Condensed Water Vapor	The Balance of All-Year Amount of Condensed and Evaporated Water Vapor	Thermal Damping Factor	Phase Shift of Thermal Oscillation
	*U*	*R_o_*	*θ* *_si_*	*g_k_*	*g_k_-g_v_*	*v*	*ψ*
	[W/m^2^·K]	[m^2^·K/W]	[°C]	[kg/m^2^·year]		-	hours
LW1	0.153	6.372	19.305	0.095	−0.178	2520.519	20.958
LW2	0.153	6.371	19.304	0.381	−0.420	557.321	20.944

Thermal performance properties were computed in accordance with the national standard STN 73 0540-2:2019 [17].

**Table 2:** Results of ANOVA for the insulation STEICO Flex width 40 mm.

Effect	Two-Way ANOVA, Moisture Content MC (%)				
	df	SS	MS	F-Test	*p*-Value
Season	1	0.0023	0.0023	64.89	0.0000
Type of Log Wall	1	0.0027	0.0027	76.04	0.0000
Season * Type	1	0.0000	0.0000	0.01	0.9318
Error	78	0.0028	0.0000		

Note: df—degree of freedom, SS—sum of squares, MS—mean square.

**Table 3:** Results of ANOVA for the insulation STEICO Universal width 22 mm.

Effect	Two-Way ANOVA, Moisture Content MC (%)				
	df	SS	MS	F-Test	*p*-Value
Season	1	0.0009	0.0009	34.58	0.000
Type of Log Wall	1	0.0001	0.0001	3.21	0.077
Season* Type	1	0.0000	0.0000	1.44	0.234
Error	78	0.0020	0.0000		

Note: df—degree of freedom, SS—sum of squares, MS—mean square.

**Table 4:** Results of ANOVA for the insulation STEICO Flex part 1 width 45 mm.

Effect	Two-Way ANOVA, Moisture Content MC (%)				
	df	SS	MS	F-Test	*p*-Value
Season	1	0.0001	0.0001	4.61	0.035
Type of Log Wall	1	0.0005	0.0005	24.22	0.000
Season * Type	1	0.0001	0.0001	5.79	0.019
Error	78	0.0017	0.0000		

Note: df—degree of freedom, SS—sum of squares, MS—mean square.

**Table 5:** Results of ANOVA for the insulation STEICO Flex part 2 width 45 mm.

Effect	Two-Way ANOVA, Moisture Content MC (%)				
	df	SS	MS	F-Test	*p*-Value
Season	1	0.0006	0.0006	6.14	0.015
Type of Log Wall	1	0.0003	0.0003	3.57	0.062
Season * Type	1	0.0000	0.0000	0.03	0.866
Error	78	0.0075	0.0001		

Note: df—degree of freedom, SS—sum of squares, MS—mean square.

**Table 6:** Results of ANOVA for the insulation STEICO Flex part 3 width 50 mm.

Effect	Two-Way ANOVA, Moisture Content MC (%)				
	df	SS	MS	F-Test	*p*-Value
Season	1	0.0071	0.0071	79.84	0.000
Type of Log Wall	1	0.0000	0.0000	0.14	0.714
Season * Type	1	0.0005	0.0005	5.53	0.021
Error	78	0.0070	0.0001		

Note: df—degree of freedom, SS—sum of squares, MS—mean square.

**Table 7:** Significance Influence (yes/no) of Analyzed Factors and Interactions on the Insulation Moisture Content.

	Insulation in the Wall LW1 a LW2	Factor and Influence Significance on Material		
		Type of Log Wall	Season	Season * Type
1.	STEICO Flex: width 40 mm	yes	yes	no
2.	STEICO Universal: width 22 mm	no	yes	no
3.	STEICO Flex part 1: width 45 mm	yes	yes	yes
4.	STEICO Flex part 2: width 45 mm	no	yes	no
5.	STEICO Flex part 3: width 50 mm	no	yes	yes

Note: Log wall type: LW1 with Vapor barrier and LW2 without Vapor barrier. Season: Heating season from 1.11. to 30.4. (6 months), non-heating season from 1.5 to 30.10 (6 months).

**Table 8:** Student T-test Results of Relative Air Humidity in the Walls in the HS21.

Relative Air Humidity in the Walls (%)	Student T-Test for Single Mean						
	Mean	St. Dev.	N	Limit Value	*t*-Test	df	*p*-Value
LW1	83.294	2.753	181	84	−3.45	180	0.000
LW2	84.002	3.300	181	84	0.01	180	0.249

Note: St. Dev.—standard deviation.

**Table 9:** Student T-test Results of Relative Air Humidity in the Walls from 1 December 2020 to 30 April 2021.

Relative Air Humidity in the Walls (%)	Student T-Test for Single Mean						
	Mean	St. Dev.	N	Limit Value	*t*-Test	df	*p*-Value
LW1	84.288	1.359	151	84	2.61	150	0.005
LW2	85.175	1.775	151	84	8.13	150	0.000

## Data Availability

Not applicable.

## Nomenclature

ANOVA	analysis of variance
df	degree of freedom
F-test	statistical hypothesis test
HS	heating season
LW1	Log Wall 1, with vapor barrier
LW2	Log Wall 2, without vapor barrier
λ	thermal conductivity coefficient (W·m^−1^·K^−1^)
MC	moisture content
MS	mean square
MSL	meters above sea level
µ	diffusion resistance factor
nHS	non-heating season
*p*-value	probability value
RH	relative humidity
Sd	equivalent diffuse thickness (m)
SS	sum of squares
T-test	Student’s statistical hypothesis test
